# Druggable Molecular Pathways in Chronic Lymphocytic Leukemia

**DOI:** 10.3390/life12020283

**Published:** 2022-02-14

**Authors:** Mohammad Almasri, Marah Amer, Joseph Ghanej, Abdurraouf Mokhtar Mahmoud, Gianluca Gaidano, Riccardo Moia

**Affiliations:** Division of Hematology, Department of Translational Medicine, Università del Piemonte Orientale, 28100 Novara, Italy; 20041440@studenti.uniupo.it (M.A.); 20041444@studenti.uniupo.it (M.A.); 20041548@studenti.uniupo.it (J.G.); abdurraouf.mahmoud@uniupo.it (A.M.M.); riccardo.moia@uniupo.it (R.M.)

**Keywords:** chronic lymphocytic leukemia, precision medicine, target therapy

## Abstract

Chronic lymphocytic leukemia (CLL), the most common type of leukemia in adults, is characterized by a high degree of clinical heterogeneity that is influenced by the disease’s molecular complexity. The genes most frequently affected in CLL cluster into specific biological pathways, including B-cell receptor (BCR) signaling, apoptosis, NF-κB, and NOTCH1 signaling. BCR signaling and the apoptosis pathway have been exploited to design targeted medicines for CLL therapy. Consistently, molecules that selectively inhibit specific BCR components, namely Bruton tyrosine kinase (BTK) and phosphoinositide 3-kinase (PI3K) as well as inhibitors of BCL2, have revolutionized the therapeutic management of CLL patients. Several BTK inhibitors and PI3K inhibitors with different modes of action are currently used or are in development in advanced stage clinical trials. Moreover, the restoration of apoptosis by the BCL2 inhibitor venetoclax offers meaningful clinical activity with a fixed-duration scheme. Inhibitors of the BCR and of BCL2 are able to overcome the chemorefractoriness associated with high-risk genetic features, including *TP53* disruption. Other signaling cascades involved in CLL pathogenesis, in particular NOTCH signaling and NF-kB signaling, already provide biomarkers for a precision medicine approach to CLL and may represent potential druggable targets for the future. The aim of the present review is to discuss the druggable pathways of CLL and to provide the biological background of the high efficacy of targeted biological drugs in CLL.

## 1. Introduction

Chronic lymphocytic leukemia (CLL) is the most common type of leukemia in adults. The median age at diagnosis is 72 years, and the male to female ratio is 1.7:1. CLL is characterized by the monoclonal expansion of mature B cells with typical phenotype (CD5+ CD19+ CD20+ CD23+ sIg low) in the peripheral blood, bone marrow, and lymphoid tissues. The development of CLL is often preceded by a non-symptomatic precursor state called monoclonal B-cell lymphocytosis (MBL), defined as a monoclonal B-cell count <5 × 10^9^/L with the typical phenotype of CLL [1].

CLL is characterized by a marked degree of heterogeneity both at the clinical and at the biological level. Some patients have an indolent disease that does not require therapy for many years. Conversely, other patients have an aggressive disease that requires treatment soon after diagnosis and/or may subsequently undergo histologic transformation into an aggressive lymphoma, known as Richter syndrome [2]. The biological heterogeneity of CLL can be ascribed to the immunogenetic origin of the disease, as reflected by immunoglobulin heavy-chain (IGHV) gene status as well as to the profile of genetic alterations of proto-oncogenes and tumor-suppressor genes that are acquired by each individual patient [2,3,4].

The mutational status of IGHV genes plays a pivotal role in the biological and clinical profile of CLL. Mutated CLL (M-CLL) displays a rate of somatic hypermutation in the IGHV genes higher than 2% when compared to the corresponding germline IGHV gene counterpart, derived from post-germinal center (GC) B cells, and generally has an indolent disease course [5]. Conversely, unmutated CLL (U-CLL) displays a rate of IGHV gene somatic hypermutation lower than 2% compared to the corresponding germline IGHV gene counterpart, does not experience the GC reaction, and displays a more aggressive disease course [6,7]. B-cell receptor (BCR) signaling plays a fundamental pathogenic role in CLL, as documented by the biased usage of IGHV genes as well as by evidence of the dependence of CLL cell survival and growth upon BCR signaling [8,9]. These notions point to the BCR as a key component in CLL development and progression and as a main druggable pathway for molecular therapy with BCR inhibitors [8].

In addition to the BCR pathway, several molecular studies have identified different genetic lesions that might be used as molecular predictors or therapeutic targets [3,4,10]. The genetic landscape of CLL lacks a unifying molecular alteration, is markedly heterogeneous, and includes gross chromosomal aberrations, namely del13q14, trisomy 12, del17p13, and de11q23 as well as mutations of many cancer-related genes. The genes most frequently affected by molecular alterations in CLL cluster into specific biological pathways, including NOTCH1 signaling (*NOTCH1* and *FBXW7*), DNA damage response (*ATM, TP53, POT1*), apoptosis (*miR15/16* and *BCL2*), BCR and toll-like receptor (TLR) signaling (*EGR2, BCOR, MYD88, TLR2, IKZF3*), NF-κB signaling (*BIRC3, NFKBIE, TRAF2, TRAF3*), and RNA splicing and metabolism (SF3B1, U1, *XPO1, DDX3X, RPS15*) (Table 1) [9,11,12].

The continuous improvement in the understanding of CLL pathogenesis has allowed the development of novel therapies that specifically target pivotal signaling pathways of CLL cells. In the present review, we cover the main biological pathways of CLL pathogenesis and the potential vulnerabilities that might be targeted in each pathway. Targeted therapy has already entered the clinical practice of CLL since several years, and its role is continuously expanding. In fact, seminal translational studies have led to understand that CLL genetic features (i.e., IGHV mutational status and *TP53* abnormalities) are important biomarkers of refractoriness to chemotherapy and, therefore, act as predictors for treatment choices [2,4,13,14]. For example, *TP53* disruption and unmutated status of IGHV genes are well-established predictors of chemorefractoriness that mandate treatment with targeted agents (BCR and/or BCL2 inhibitors) that can circumvent, at least in part, the CLL refractoriness to chemo-immunotherapy (CIT). In addition, other gene mutations (i.e., *NOTCH1*, *BIRC3*) are under scrutiny in order to clearly define their prognostic and/or predictive value [3,10,15,16,17].

## 2. Targeting the BCR in CLL

**Figure 1:**
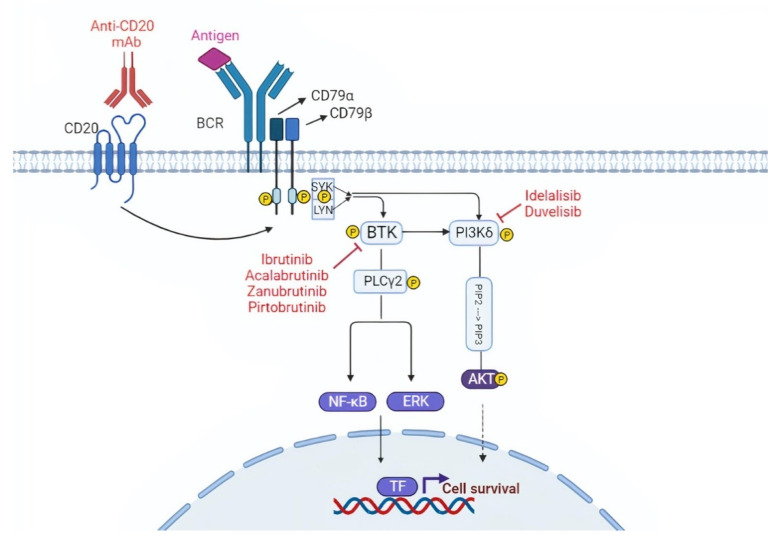
Targeting the B cell receptor (BCR) signaling pathway. The BCR is constituted of a membrane immunoglobulin attached to the CD79a/CD79b complex. The antigen binding leads to the interaction between the ITAM domain of CD79a/CD79b and the Syk and Lyn kinases. This interaction triggers the downstream BCR signaling cascade. BTK and PI3K play a pivotal role in the BCR cascade and drugs that inhibit these two molecules are represented in the figure. TF, transcription factors.

The BCR consists of a membrane immunoglobulin non-covalently bound to a heterodimer composed of CD79α (Igα) and CD79β (Igβ). In normal B cells, antigen binding to the BCR triggers the downstream signaling cascade, thus inducing cell proliferation, survival, and differentiation stimuli (Figure 1) [18,19]. Compared to normal B cells, the BCR of many CLL cells is characterized by an intrinsically higher reactivity to antigens [20,21]. Moreover, in some cases, the BCR of CLL cells may also interact with a BCR expressed on other CLL cells, thus auto-enhancing BCR signaling [21,22]. About one-third of patients with CLL carry quasi-identical BCR sequences that can be classified into stereotyped BCR subsets based on the structure of their complementarity-determining regions (CDRs) [23,24]. Approximately 200 different CLL stereotyped BCR subsets have been identified to date [23]. These findings reinforce the notion that specific antigenic stimuli triggering the BCR may be involved in CLL pathogenesis.

The BCR is connected to a network of kinases and phosphatases that regulate and amplify its activation. Upon antigen binding, the BCR initiates a signaling cascade through the phosphorylation of Igα (CD79α) and Igβ (CD79β) by Lyn and other Src family kinases. These events are followed by the activation of other kinases, namely SYK, Bruton tyrosine kinase (BTK), and phosphoinositide-3 kinases (PI3Ks), which will transmit the signal to downstream pathways important for B-cell growth and survival, including AKT, ERK, and NF-κB (Figure 1) [19].

As stated above, BCR signaling is essential for CLL pathogenesis and proliferation and can be targeted in vivo by inhibiting the BTK that plays a pivotal role in the BCR cascade. Different BTK inhibitors (BTKi) with different modes of action are currently used or are in development in advanced stage clinical trials. In addition to BTK, PI3K is also a druggable target in CLL.

### 2.1. BTK Inhibitors

BTK is a crucial intracellular protein downstream of the BCR, whose expression is upregulated in CLL cells [25]. BTKi are small, orally available molecules that bind to the Cys481 residue near to the ATP-binding domain of the BTK protein by covalent or non-covalent bonds. This active occupancy of the ATP binding domain inhibits the subsequent phosphorylation of BTK and blocks the downstream signaling pathways, including AKT and NF-kB, which regulate cell survival and proliferation. BTKi can be grouped into covalent BTKi (ibrutinib, acalabrutinib, and zanubrutinib) and non-covalent BTKi (pirtobrutinib) [26,27].

CLL patients may develop resistance against BTKi by different mechanisms, including mutations of the BTK binding site and of the gene encoding phospholipase C Gamma 2 (PLCG2), which acts downstream of BTK in the BCR signaling cascade. BTK mutations are represented by substitutions of the Cys481 residue with a different amino acid, leading to the loss of the bond between the drug and the kinase. PLCG2 alterations are gain of function mutations, which can activate downstream BCR signaling independent of BTK inhibition. Both BTK mutations and PLCG2 mutations lead to loss of the activity of the BTKi [25,28,29].

#### 2.1.1. Ibrutinib

Ibrutinib, the first-in-class covalent BTKi, has significantly changed the natural history and the management of CLL patients [25]. Ibrutinib can induce off-target effects by inhibiting other kinases that have a corresponding cysteine residue in the ATP binding site similar to BTK, such as epidermal-derived growth factor receptor (EGFR) family kinases, TEC family proteins, and interleukin-2-inducible tyrosine kinase (ITK). These off- target inhibition cause undesired side effects, which might limit the treatment, such as atrial fibrillation (AF) and bleeding [30,31].

Long-term outcomes of pivotal early phase studies have demonstrated durable responses with progression-free survival (PFS) rates that at 7 years exceed 80% in treatment-naïve patients [32]. Importantly, these reports highlight that ibrutinib is highly active also in *TP53*-disrupted patients, in which the drug allows to achieve a 6-year PFS and overall survival (OS) of 61% and 79%, respectively [33]. Subsequent phase 3 trials of ibrutinib single agent or in combination with anti-CD20 monoclonal antibodies (mAb) have shown that the drug significantly prolongs outcomes of both young and fit CLL patients and elderly patients with comorbidities compared to CIT regimens [34,35,36]. Interestingly, ibrutinib completely overcomes the negative prognostic impact of unmutated IGHV genes, and since its mode of action is independent of *TP53*, it also smoothens the detrimental impact of *TP53* disruption in CLL cells [37] (Table 2).

#### 2.1.2. Acalabrutinib

Acalabrutinib is a more potent and selective inhibitor of BTK in comparison to ibrutinib, and given the lower activity of the drug against other kinases (e.g., ITK, EGFR, ERBB2), it is less likely to cause off-target adverse events [44,45]. Acalabrutinib has demonstrated superior PFS compared to CIT or to the PI3Kδ inhibitor idelalisib in a phase 3 study dedicated to relapsed/refractory (R/R) patients (ASCEND trial) [46]. Furthermore, acalabrutinib single agent or in combination with the anti-CD20 mAb Obinutuzumab has been shown to prolong PFS in first-line setting in elderly CLL patients with comorbidities (ELEVATE-TN trial) [42]. Because of its higher affinity for BTK, acalabrutinib has also demonstrated high efficacy and tolerability in ibrutinib-intolerant patients with CLL [47,48].

Recently, a head-to-head comparison of ibrutinib versus acalabrutinib has been carried out in a phase 3 trial dedicated to R/R CLL patients (ELEVATE-RR). This trial has demonstrated the non-inferiority of acalabrutinib compared to ibrutinib in terms of PFS and has documented that acalabrutinib associates with an improved safety profile with fewer AF events and discontinuations because of adverse events [41] (Table 2).

#### 2.1.3. Zanubrutinib

Zanubrutinib is a next-generation BTKi with favorable oral bioavailability and high specificity for BTK, exhibiting lower off-target activity than ibrutinib for structurally related kinases, such as EGFR and ITK [49]. Phase 2 studies in both the treatment naïve (TN) and R/R CLL with *TP53* disruption have shown an overall response rate (ORR) of more than 85% [50,51,52]. Recently, an interim analysis of a phase 3 randomized, controlled trial comparing ibrutinib versus zanubrutinib in R/R CLL patients has demonstrated that zanubrutinib has a superior response rate, an improved PFS, and a lower rate of atrial fibrillation/flutter compared to ibrutinib [53].

#### 2.1.4. Pirtobrutinib

The most common mechanisms of resistance to covalent BTKi include mutations at the binding site of the drugs (BTK Cys481) and gain-of-function mutations of the downstream PLCγ2 phospholipase [54,55]. Pirtobrutinib is an orally available, highly selective, reversible BTKi with equal low nM potency against both wild-type and Cys481-mutated *BTK* [56]. In a phase 1/2 study of pirtobrutinib in B-cell malignances, the ORR of R/R CLL patients to pirtobutinib was 62% [57]. The ORR was similar in CLL patients who had been exposed and become resistant to covalent BTKi, had developed intolerance to covalent BTKi, had acquired a Cys481 mutation in the *BTK* gene, or had a BTK wild-type disease [57]. In terms of side effects, pirtobrutinib demonstrated a good safety profile. In fact, grade 3 AF or flutter was not observed, and only 1% of patients discontinued treatment due to a therapy-related adverse event [57].

### 2.2. PI3K Inhibitors

PI3Ks are a family of enzymes involved in cellular functions, such as cell growth, proliferation, differentiation, and survival, and are frequently dysregulated in cancers. PI3K are subdivided into three classes, termed as class I, II, and III. Class I PI3Ks comprise four isoforms, namely PI3K-α, -β, -γ, and -δ. The PI3Kδ isoform is a kinase that amplifies and transduces signals from the BCR on the cell surface to the downstream AKT signaling pathway and is the most relevant target in CLL [12,58]. PI3Ki are small, orally available molecules that bind the ATP binding pocket of the PI3K. As a consequence, a major survival signaling pathway in CLL cells, involving AKT, will be inhibited [58]. In addition to PI3Ks inhibitors, this pathway can also be inhibited by the AKT o mTOR inhibitors that are in development [59].

PI3Ki molecules have various specificities and affinities to bind the different PI3K isoforms. One of the major challenges in the development of PI3Ki is the inability to achieve an optimal molecule that can target a specific isoform. The toxicities from these small-molecules depend on their degree of PI3K isoform specificity [60]. As previously described, different PI3K isoforms are present in human cells, and the isoform δ is a suitable target in CLL cells [61]. Two PI3Ki have been tested in advanced stage clinical trials, namely idelalisib and duvelisib.

#### 2.2.1. Idelalisib

Idelalisib is the first commercially approved PI3Kδ inhibitor for patients with CLL. This molecule demonstrated meaningful clinical activities in R/R patients in the context of phase 2 clinical trials [62]. The phase 3 trial compared idelalisib-rituximab with placebo-rituximab in R/R CLL [63,64]. Idelalisib-rituximab resulted in superior PFS (not reached vs. 5.5 months) and OS at 12 months (92% vs. 80%) [63,64]. Patients with high-risk disease features, such as *TP53* aberrations or IGHV unmutated status, had similar PFS to those without such features [63,64]. Adverse events included neutropenia (65%), transaminitis (39%), and diarrhea or colitis in 36% of patients. Opportunistic *P jirovecii* infections were seen in patients who had not received prophylaxis [63,64].

#### 2.2.2. Duvelisib

The efficacy of idelalisib encouraged development of other PI3Ki. Duvelisib is an oral dual inhibitor of PI3Kd and PI3Kg that is uniquely positioned to target both intracellular and extracellular survival signals [65]. Considering all tested dose levels, adverse events were similar to those reported for idelalisib, with neutropenia (39%), increased transaminase levels (39%), and diarrhea (42%). Early efficacy data in CLL were promising, with a 56% ORR and a median PFS of 15.7 months across all dose levels [66]. The subsequent phase 3 trial tested duvelisib versus ofatumumab (an anti-CD20 monoclonal antibody) in R/R CLL. The median PFS was superior for duvelisib versus ofatumumab (17.6 months versus 9.7 months) [43]. As with idelalisib, PFS was similar in *TP53*-aberrant and *TP53*-intact disease [43]. Adverse events were similar to those reported in earlier phase trials, and cases of *P jirovecii* pneumonia were restricted only to subjects not receiving anti-infectious prophylaxis [43,67] (Table 2).

## 3. Targeting the *BCL2* Pathway in CLL

**Figure 2:**
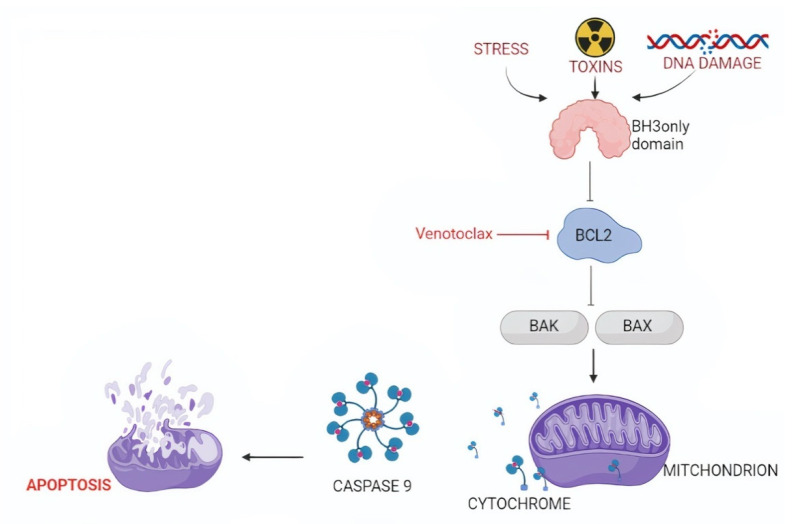
Targeting the intrinsic pathway of apoptosis. The intrinsic pathway of apoptosis is activated by diverse cytotoxic stimuli, including oncogenic stress and chemotherapeutic agents. These pro-apoptotic factors trigger BH3-only proteins, including BIM, to bind and inhibit BCL2. As a consequence of BCL2 inhibition, larger amounts of BAK and BAX will be rendered free. The availability of large amounts of BAK and BAX allows them to dimerize and create a channel for cytochrome c leakage from the mitochondria into the cytoplasm, where it induces cell apoptosis.

The BCL-2 family contains different members that can be divided into two groups: (i) pro-apoptotic proteins, including multi-domain (e.g., BAK and BAX) or BH3-only proteins (e.g., BIM and PUMA) and (ii) anti-apoptotic proteins, namely BCL2, BCL-X_L_, BCL-W, MCL1, BCL-B, and BFL-1 [67,68]. Physiologically, there is a balance between pro-apoptotic and anti-apoptotic actors. BCL2 inhibits apoptosis by sequestering BH3-only proteins that are required for the activation of BAK and BAX (Figure 2) [68,69].

CLL cells consistently overexpress BCL2, rendering it an important druggable target. Del13q14, the most common genetic abnormality in CLL, occurs in at least 40–50% of cases and is a well-documented mechanism of BCL2 deregulation in CLL. Seminal studies identified two microRNAs, termed *miR-15* and *miR-16*, as the relevant genes that are lost because of del13q14 [70]. By binding to specific sequences on *BCL2* mRNA, *miR-15* and *miR-16* inhibit the translation of the BCL2 protein. In case of del13q14, the function of *miR-15* and *miR-16* is lost, and the translation of *BCL2* mRNA is no longer inhibited. Consequently, translation of the BCL2 protein will be enhanced, the cellular levels of BCL2 expression will increase, apoptosis will be prevented, and survival of CLL cells will be promoted [71,72].

### BCL2 Inhibitors

Given the pivotal role of BCL2 in the pathogenesis of CLL, different molecules have been designed to target BCL2 in this leukemia. The first BCL2 inhibitor (BCL2i) to be tested in humans was navitoclax [72], which, however, did not selectively inhibit BCL2 but also targeted BCL-X_L_. Navitoclax demonstrated meaningful clinical activity in CLL, but its usage was limited by the occurrence of thrombocytopenia regardless of the dose because of the inhibition of BCL-X_L_, which is a pro-survival protein relevant for circulating platelets [73]. The search for novel BCL2i subsequently led to the design of venetoclax, which is currently used in hematological malignancies.

Venetoclax is a BH3-mimetic molecule that binds to BCL2 similarly to BH3-only proteins, in particular BIM and BID, but with higher affinity. Consequently, venetoclax inhibits the BCL2 ability to bind and quench BIM and BID. Thus, BIM and BID can interact with BAX and BAK and activate the intrinsic apoptotic cascade. Venetoclax is more specific for BCL2 than for BCL-X_L_, and few events of thrombocytopenia have been noted. [74].

The first in-human phase 1 trial of venetoclax in CLL enrolled 116 patients. One of the main adverse events was tumor lysis in the first three patients due to the high efficacy of this molecule in inducing apoptosis [75]. This event led to the introduction of a stepwise intra-patient increase in venetoclax dosage (the so-called ramp-up) and the prophylaxis of tumor-lysis syndrome (TLS). Venetoclax showed great efficacy with 79% ORR and 20% complete response (CR) [75]. The subsequent phase 2 study enrolled 107 R/R CLL with 17p deletion. The ORR was 79%, and patients with a high proportion of cells with 17p deletion or with *TP53* mutations experienced superimposable outcomes [76]. In another phase 2 trial, venetoclax showed high efficacy also in patients who relapsed after ibrutinib with a PFS and OS at 12 months of 75% and 91%, respectively [77].

Based on the results obtained in monotherapy and given the synergism of venetoclax with different molecules, venetoclax has also been used in combination with other drugs in different clinical trials. Initial phase 1b and 2 studies showed high efficacy of venetoclax in combination with both anti-CD20 antibodies and BTKi [78,79]. These results prompted the design of the phase 3 MURANO trial comparing bendamustine-rituximab versus fixed-duration venetoclax-rituximab (6 doses of rituximab and 2 years of venetoclax) in R/R CLL. Venetoclax-rituximab significantly improved PFS and OS of CLL patients and was therefore approved for R/R CLL patients [40]. Venetoclax was also combined with the second-generation anti-CD20 mAb Obinutuzumab in elderly CLL patients with comorbidities. The CLL14 trial compared venetoclax-Obinutuzumab versus chlorambucil-Obinutuzumab [38]. The treatment duration in both groups consisted of 12 cycles lasting 28 days each. The PFS at 24 months was significantly higher in the venetoclax-Obinutuzumab group than in the chlorambucil-Obinutuzumab group: 88.2% compared with 64.1% (*p* < 0.001). This benefit was also observed in patients with *TP53* deletion, mutation, or both, and in patients with unmutated IGHV genes [38,80,81].

Results from preclinical studies have pointed to a potential synergy between ibrutinib and venetoclax. In fact, among the anti-apoptotic proteins that are overexpressed in CLL, levels of the anti-apoptotic MCL1 and BCL-X_L_ proteins are decreased after ibrutinib, while venetoclax selectively antagonizes BCL2 [82,83]. The phase 3 GLOW study compared ibrunitib-venetoclax with chlorambucil-Obinutuzumab in elderly CLL patients with comorbidities. Ibrutinib-venetoclax significantly improved PFS compared to chlorambucil-Obinutuzumab with manageable toxicities [84] (Table 2).

## 4. Targeting Notch Signaling in CLL

**Figure 3:**
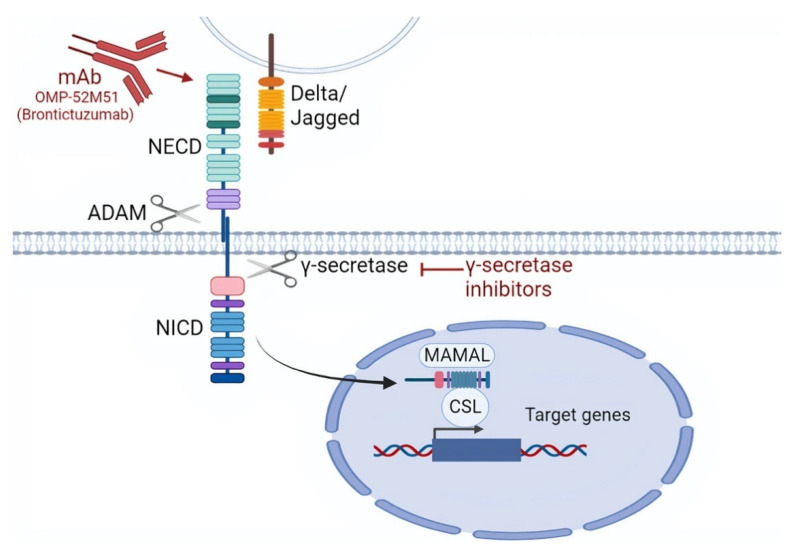
Targeting the NOTCH signaling pathway. NOTCH signaling can be targeted by monoclonal antibodies mAbs that are directed against the NOTCH extra cellular domain (NECD). Other mechanisms of NOTCH inhibition include the targeting by γ-secretase inhibitors that block γ-secretase, thus inhibiting the cleavage of NICD, which is necessary for nuclear translocation. As a consequence, γ-secretase inhibitors prevent the transcriptional activation of NOTCH1 target genes through the suppression of the MAML (Mastermind-like) and CSL (CBF1, Suppressor of Hairless, Lag-1) transcription factors.

The Notch signaling pathway is composed of a family of transmembrane receptors, of which four are present in humans, namely *NOTCH1*, *NOTCH2*, *NOTCH3*, and *NOTCH4* [85]. In normal cells, Notch signaling is activated by cell-to-cell contact thanks to the interaction between the Notch extracellular domain (NECD) and a ligand belonging to members of the Delta-like (DLL1, DLL3, DLL4) or Jagged family (JAG1, JAG2). This receptor-ligand interaction triggers two sequential proteolytic cleavages in Notch receptors by A Disintegrin and Metalloproteinase domain-containing protein (ADAM) 10/17 and γ-secretase, which generate three domains, namely (i) NECD; (ii) Notch transmembrane domain (NTMD); and (iii) Notch intracellular domain (NICD) [85,86]. The NICD then translocates to the nucleus, associates with the DNA-binding factor RBPJ, and positively regulates gene transcription. The NICD is usually short-lived because the C-terminal portion, known as PEST domain, is recognized by an E3 ubiquitin ligase and degraded (Figure 3) [85].

In CLL, *NOTCH1* is altered in approximately 10–15% of patients [11,12]. Most of *NOTCH1* mutations disrupt the PEST domain that is essential for NOTCH1 proteasomal degradation. Consequently, NOTCH1 is no longer ubiquitinated, and consequently, transcription of NOTCH1 target genes is constitutively deregulated. NOTCH1 signaling may also be enhanced by mutations of *FBXW7*, a gene coding for a NOTCH1 ubiquitinase, whose disruption impairs the ubiquitination of the NOTCH1 protein. Rarely, point mutations in the 3′UTR of the NOTCH1 mRNA lead to aberrant splicing events that cause the loss of the NOTCH1 PEST domain [85,87,88].

*NOTCH1* mutations associate with shorter survival compared to wild-type patients when treated with CIT. Interestingly, results from the CLL8 trial comparing fludarabine, cyclophosphamide, rituximab (FCR) versus fludarabine, and cyclophosphamide (FC) as first line therapy in CLL patients demonstrated that *NOTCH1* mutated patients may not benefit from the addition of the type 1 anti-CD20 mAb rituximab. Conversely, the novel type 2 anti-CD20 mAb Obinutuzumab appears to overcome the refractoriness to anti-CD20 therapy in CLL carrying *NOTCH1* mutations [89].

Different studies are exploring potential molecules with different modes of action that may inhibit the NOTCH signaling pathway, including (i) mAbs against a fragment of the human NOTCH1 protein and (ii) γ secretase inhibitors. The mAb OMP-52M51 (Brontictuzumab) has been shown to efficiently block canonical Notch signaling and to decrease Notch activation also in the presence of PEST mutations in vitro [90]. This drug was tested in a phase 1 dose escalation trial (NCT01778439) in patients with previously treated CLL, mantle cell lymphoma, T-lineage acute lymphoblastic leukemia (T-ALL), or other hematologic malignancies but showed limited antitumor efficacy [91]. Concerning γ-secretase inhibitors, these compounds have been tested in T-ALL showing cell cycle arrest and rapid clearance of intracellular NOTCH1 [85,92]. In CLL, however, the activity of γ-secretase inhibitors has not been documented.

Although BCRi and BCL2i do not target NOTCH1 directly, these drugs appear to circumvent the negative prognostic impact of *NOTCH1* mutations conferred to patients treated with CIT. In this respect, the clinical impact of *NOTCH1* mutations in patients treated with BCRi or BCL2i has been addressed by two large prospective studies. Results from the RESONATE and CLL14 clinical trials indicate that arms containing ibrutinib (in the case of RESONATE) or venetoclax (in the case of CLL14) are able to overcome the negative prognostic impact conferred by *NOTCH1* mutations [37,80].

## 5. Targeting the NF-κB Signaling Pathway in CLL

**Figure 4:**
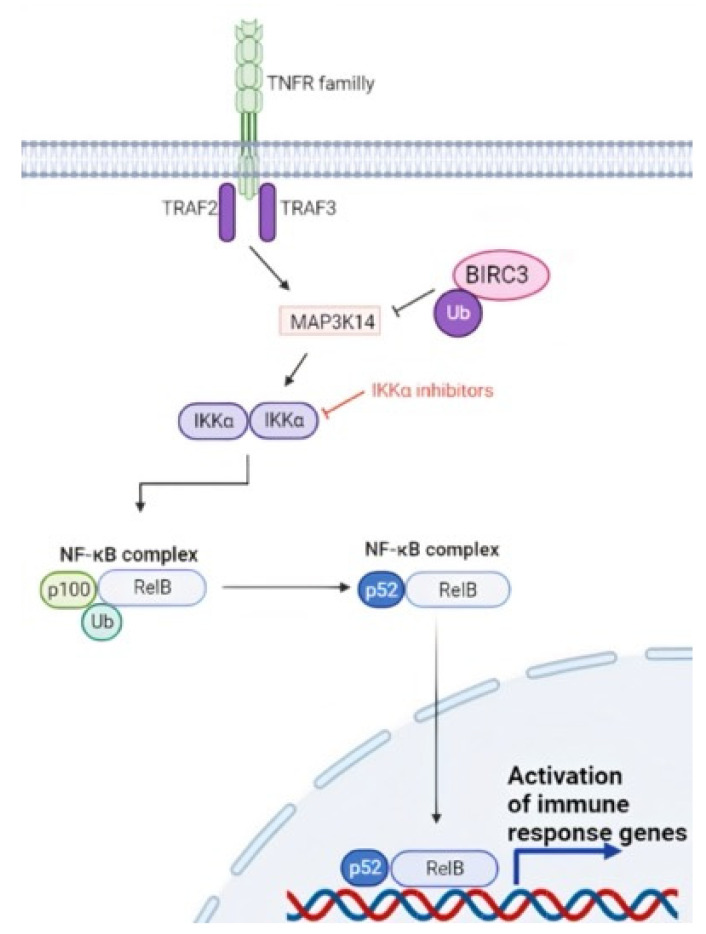
Non canonical NF-κB signaling in CLL. BIRC3 is a negative regulator of the non-canonical NF-κB pathway, and the *BIRC3* gene is disrupted by loss of function genetic alterations in a fraction of CLL. BIRC3 physiologically catalyzes the ubiquitination of MAP3K14, leading to inactivation of the NF-κB pathway. In the case of *BIRC3*-disrupting mutations, MAP3K14 is no longer ubiquitinated, and therefore, MAP3K14 can perform its function of positive signal transducer activating the NF-κB pathway.

Nuclear factor-κB (NF-κB) signaling is a key component of the development and evolution of CLL. Two NF-κB pathways exist, namely the canonical and the non-canonical pathways. The canonical pathway is mediated primarily by signals originating from cell surface receptors, such as the BCR, and is activated by the IkB-kinase (IKK) complex [93]. Activation of the IKK complex leads to phosphorylation and subsequent ubiquitin-mediated proteasomal degradation of the inhibitor of NF-κB proteins (IkBs) [93]. The non-canonical pathway is activated by members of the tumor necrosis factor (TNF) cytokine family. Upon receptor binding, the TRAF3/MAP3K14-TRAF2/BIRC3-negative regulatory complex of non-canonical NF-κB signaling is disrupted. As a consequence, MAP3K14, the central activating kinase of the pathway, is released to induce the phosphorylation and proteasomal processing of p100, thereby leading to the formation of p52-containing NF-κB dimers. The p52 protein subsequently dimerizes with RelB and translocates into the nucleus, where it regulates gene transcription (Figure 4) [94].

In CLL, the NF-kB signaling pathway may be deregulated by mutations affecting the *BIRC3* gene, which encodes for a component of the TRAF3/MAP3K14-TRAF2/BIRC3-negative regulatory complex of non-canonical NF-κB signaling [9]. Virtually all *BIRC3* genetic lesions are frameshift mutations or stop codons clustering in two hotspot regions between amino acids 367–438 and 537–564. *BIRC3* variants are predicted to generate aberrant truncated transcripts that truncate the C-terminal RING domain of BIRC3. The RING domain of BIRC3 harbors the E3 ubiquitin ligase activity that is essential for proteasomal degradation of MAP3K14, the central activating kinase of non-canonical NF-κB signaling. This observation points to non-canonical NF-κB activation through MAP3K14 stabilization as the predicted functional consequence of *BIRC3* mutations in CLL. *BIRC3* is mutated in approximately 3–4% of newly diagnosed CLL and in 25% of chemo-refractory patients [17,94,95]. In addition, recent works evaluated the impact of biallelic *BIRC3* loss in CLL cases harboring 11q deletions showing that *BIRC3* mutations in del(11q) cells promote clonal advantage in vitro and accelerate leukemic progression [96].

As a prognostic biomarker, *BIRC3* mutations significantly associate with shorter PFS in FCR-treated CLL. Additionally, patients with a biallelic disruption of *BIRC3* have a shorter time to first treatment when compared to *BIRC3* wild-type patients [17,96]. Molecular analysis of the MURANO trial dedicated to R/R CLL has further documented the poor prognosis conferred by *BIRC3* disruption in CLL patients treated with CIT. In fact, *BIRC3*-mutated patients treated with bendamustine-rituximab experienced a worse outcome compared to wild-type patients. Conversely, in the same trial, the combination of the BCL2i venetoclax with rituximab was able to overcome the negative impact of *BIRC3* mutations [97]. Similarly, the molecular analysis of the CLL14 trial indicated that the combination of a BCL2i and an anti-CD20 mAb, i.e., Obinutuzumab-venetoclax, but not the Obinutuzumab-chlorambucil CIT regimen is an effective therapeutic option for *BIRC3*-mutated patients [80]. *BIRC3* is thus emerging as a novel predictive biomarker that might enter the routine clinical practice in the future.

Although direct inhibitors of the NF-κB signaling pathway are not yet available for CLL treatment, the MURANO and the CLL14 trials document that BCL2 inhibition may circumvent and overcome the activation of NF-κB that is due to *BIRC3* disruption in CLL. This observation may be ascribed to the fact that NF-κB signaling induces BCL2 transcription and expression, thus providing a suitable target for the BCL2i venetoclax.

## 6. Targeting Molecular Pathways in High-Risk CLL with TP53 Disruption

In CLL cells with dysfunctional *TP53*, DNA damage cannot induce cell cycle arrest or DNA repair, enabling the accumulation of substantial levels of DNA alterations that increase genomic instability and thereby lead to the emergence of subclones with additional genetic mutations that can drive CLL progression and transformation [98]. In CLL, the prevalence of *TP53* abnormalities, including del17p and *TP53* mutations, varies across the different phases of the disease. In early stage asymptomatic CLL patients, *TP53* abnormalities are detected in approximately 5–7% of cases [2,99,100,101,102]. The frequency of *TP53* disruption rises to 40% in fludarabine-refractory patients and to 60% in patients with Richter syndrome.

*TP53* gene defects represent a key decision-making biomarker in the algorithm for CLL treatment. In fact, del17p13 and *TP53* mutations consistently associate with adverse disease outcome in patients treated with CIT due to chemorefractoriness [2,13,103]. Assessment of *TP53* aberrations is mandatory for patients requiring therapy and must be retested before the initiation of any subsequent line of therapy since clonal evolution can occur [13,98,104]. Due to the chemorefractoriness imposed by *TP53* disruption, the status of *TP53* represents the pivotal decisional node for treatment tailoring in CLL and prompts upfront treatment with BCRi and BCL2i that, at least in part, circumvent *TP53*-mediated chemorefractoriness. However, both in patients treated with BCRi and with BCL2i, *TP53* disruption remains a negative prognostic factor. This concept is important especially in *TP53*-disrupted patients treated with fixed-duration regimens with BCL2i, indicating the need for more prolonged or continuous treatment in this genetic subset of patients [4].

BCRi and BCL2i do not directly target the TP53 protein but instead exert their antileukemic action through a TP53 independent pathway. Different strategies are currently ongoing with the aim of directly targeting the TP53-dysfunctional protein. APR-246 selectively induces apoptosis in cancer cells with mutant *TP53*. Mechanistically, APR-246 is able to restore the physiological TP53 conformation and function in cell lines with *TP53* missense mutations [105,106]. Significant results have been obtained also in vivo in myelodysplastic syndromes and acute myeloid leukemia although results in the CLL are still lacking.

## 7. Perspectives

CLL is the most common type of leukemia in the adult population, and 0.6% of patients at some point during their lifetime will receive a CLL diagnosis [107]. The recent advances in the understanding of the biology of the disease have allowed the identification of several critical pathways involved in CLL pathogenesis. This has enabled the subsequent design of molecules, namely BTKi and BCL2i, that selectively target at least some of these pathways and changed the therapeutic scenario of CLL patients [4]. In addition, the combination of molecules that inhibit both the BCR and the BCL2 pathway showed and event further activity [84]. In the current therapeutic landscape of CLL, which ranges from CIT to mAb and target therapy, the molecular status of IGHV and *TP53* genes provides robust predictors for treatment decision making and must be routinely assessed in the clinical practice in cases requiring treatment [13]. Novel genetic (e.g., *BIRC3* and *NOTCH1* mutations) and immunogenetic (e.g., stereotyped BCR subset #2) biomarkers are emerging and have the potential to enter the clinical practice in the future [4]. One important aspect that requires further investigations in the future is the analysis of the genetic complexity and diversity of different anatomical compartments of the disease, mainly represented by the lymph node, bone marrow, and peripheral blood compartments. A recent study in small lymphocytic lymphoma, a disease closely related to CLL, has highlighted that some biological pathways, e.g., NF-kB, may be genetically altered in only one specific anatomical compartment and not in all [108]. This spatial and biological heterogeneity may have important therapeutic implications when targeting specific biological pathways for CLL treatment. Another important topic that will need further investigation is represented by the pivotal role in CLL pathogenies of the interaction between neoplastic B cells and the tissue microenvironment [109].

In the era of precision medicine, CLL is continuing to represent an important disease model in which the molecular and clinical characteristics of the individual patient guide treatment choices. The continuing improvement of the understanding of CLL pathogenesis, coupled with the development of more specific targeted medicines for B-cell malignancies that can spare off-target toxicities, represent an avenue for further improving patients’ outcomes [110].

## Figures and Tables

**Table 1:** Main molecular pathways involved by gene mutations in CLL.

The Biological Pathways	Mutated Genes
NOTCH1 Signaling	*NOTCH1, FBXW7*
BCR and Toll-like receptor signaling	*EGR2, BCOR, MYD88, TLR2, IKZF3*
MAPK-ERK pathway	*KRAS, NRAS, BRAF, MAP2K1*
RNA Splicing and metabolism	*SF3B1, U1, XPO1, DDX3X, RPS15*
NF-κB Signaling	*BIRC3, NFKBIE, TRAF2, TRAF3*
DNA damage response	*ATM, TP53, POT1*
Apoptosis	*miR15/16, BCL2*

**Table 2:** Clinical trials in CLL.

Trial	Phase	Setting	Interventions	N. of Patients	PFS	OS
Ibrutinib-Rituximab or Chemoimmunotherapy for Chronic Lymphocytic Leukemia [34]	3	Untreated patients with CLL or SLL subtype of CLL	Ibrutinib-Rituximab	354	3 years: 89.4%	3 years: 98.8%
			Chemoimmunotherapy (FCR)	175	3 years: 72.9%	3 years: 91.5%
Venetoclax and Obinutuzumab in Patients with CLL and Coexisting Conditions [38]	3	Untreated patients with CLL	Venetoclax + Obinutuzumab	216	24 months: 88.2%	24 months: 91.8%
			Chlorambucil + Obinutuzumab	216	24 months: 64.1%	24 months: 93.3%
Ibrutinib Regimens versus Chemoimmunotherapy in Older Patients with Untreated CLL [36]	3	Untreated patients with CLL aged ≥65	Bendamustine + Rituximab	183	24 months: 74%	24 months: 95%
			Ibrutinib	182	24 months: 87%	24 months: 90%
			Ibrutinib + Rituximab	182	24 months: 88%	24 months: 94%
Ibrutinib plus obinutuzumab versus chlorambucil plus obinutuzumab in first-line treatment of chronic lymphocytic leukaemia (iLLUMINATE): a multicentre, randomised, open-label, phase 3 trial [39]	3	Untreated patients with CLL or SLL either aged 65 years or older or younger than 65 years with coexisting conditions	Ibrutinib + Obinutuzumab	113	Median PFS: not reached (Estimated) 30 months: 79%	Median OS: not reached (Estimated) 30 months: 86%
			Chlorambucil + Obinutuzumab	116	Median PFS: 19 months (Estimated) 30 months: 31%	Median OS: not reached at (Estimated) 30 months: 85%
Long-term follow-up of the RESONATE phase 3 trial of Ibrutinib vs. Ofatumumab [37]	3	Previously treated patients with CLL or SLL requiring a new therapy and not eligible for purine analog-based therapy	Ibrutinib	195	Median PFS: not reached 3 years: 59%	Median OS: not reached 3 years: 74%
			Ofatumumab [Note: 68% of patients in this arm crossing over to ibrutinib]	196	Median PFS: 8.1 months 3 years: 3%	Median OS: not reached 3 years: 65%
Venetoclax-Rituximab in Relapsed or Refractory Chronic Lymphocytic Leukemia [40]	3	Patients aged 18 years or older with relapsed or refractory CLL	Venetoclax + Rituximab	194	2 years overall: 84.9%	2 years overall: 91.9%
			Bendamustine + Rituximab	195	2 years overall: 36.3%	2 years overall: 86.6%
Acalabrutinib Versus Ibrutinib in Previously Treated Chronic Lymphocytic Leukemia: Results of the First Randomized Phase III Trial [41]	3	Patients with previously treated CLL with centrally confirmed del(17)(p13.1) or del(11)(q22.3)	Ibrutinib	265	Median PFS: 34.8 months	Median OS: not reached 3 years: >60%
			Acalabrutinib	268	Median PFS: 34.8 months	Median OS: not reached 3 years: >60%
Acalabrutinib with or without obinutuzumab versus chlorambucil and Obinutuzumab for treatment-naive chronic lymphocytic leukaemia (ELEVATE TN): a randomised, controlled, phase 3 trial [42]	3	Untreated patients with CLL ged 65 years or older, or older than 18 years and younger than 65 years with creatinine clearance of 30–69 mL/min or Cumulative Illness Rating Scale for Geriatrics score greater than 6.	Acalabrutinib	179	Median PFS not reached	Median OS: not reached 3 years: >80%
			Acalabrutinib + Obinutuzumab	179	Median PFS not reached	Median OS: not reached 3 years: >80%
			Chlorambucil + Obinutuzumab	177	Median PFS: 22.6 months	Median OS: not reached 3 years: >80%
The phase 3 DUO trial: duvelisib vs. ofatumumab in relapsed and refractory CLL/SLL	3	Relapsed or refractory CLL/SLL [43]	Duvelisib	160	Median PFS: 13.3 months	Median OS: not reached 3 years: >50%
			Ofatumumab	159	Median PFS: 9.9 months	Median OS: not reached 3 years: >50%

## Data Availability

Not applicable.
